# Rectal diameter assessment in enuretic children—exploring the association between constipation and bladder function

**DOI:** 10.1080/03009734.2018.1488778

**Published:** 2018-09-04

**Authors:** Emil Jansson, Tryggve Nevéus

**Affiliations:** a Department of Internal Medicine, Avesta Hospital, Avesta, Sweden;; b Department of Women’s and Children’s Health, Uppsala University, Uppsala University Children’s Hospital, Uppsala, Sweden

**Keywords:** Constipation, detrusor overactivity, enuresis, rectal diameter, voiding chart

## Abstract

**Objectives:** Detrusor overactivity and constipation often co-exist in children with enuresis. Constipation is known to be linked to detrusor overactivity. The voiding chart is the best non-invasive way to investigate bladder function, whereas the ultrasonographical detection of rectal dilatation is the best way to objectify constipation. We wanted to investigate a possible relationship between the rectal diameter and voiding chart data in enuretic children.

**Methods:** Children with therapy-resistant enuresis were retrospectively evaluated. All had completed a voiding chart for at least 48 h. The rectal diameter was assessed ultrasonographically. The cutoff for rectal dilatation was set at 30 mm.

**Results:** We evaluated 74 patients (12 girls) aged 10.2 ± 2.8 years, 35 of whom had rectal dilatation. No significant differences in voiding chart parameters were found between children with normal versus dilated rectum. Neither did urgency or a history of daytime incontinence differ between the groups. Boys were more likely to have rectal dilatation than girls (*p* = 0.02).

**Conclusions:** The absence of differences regarding voiding chart data may be explained as two mechanisms neutralizing each other: behavioral factors may make the constipated children void seldom and with large volumes, whereas detrusor overactivity caused by rectal compression of the bladder may have the opposite effect. Another option may be that the voiding chart is too blunt an instrument to detect detrusor overactivity. Constipation, and thus presumably bladder dysfunction, seems to be more important in enuretic boys than girls.

## Introduction

Enuresis and constipation are both very common childhood conditions. Approximately 5–10% of 7-year-olds wet their beds (1), whereas the prevalence of constipation in the same age group is even greater (2). The overlap between the two conditions is greater than can be explained by chance alone (3).

Enuresis is usually caused by combinations of nocturnal polyuria, detrusor overactivity, and/or high arousal thresholds (4), detrusor overactivity being especially common among children resistant to first-line antienuretic therapy (5). Although enuresis is not primarily a psychiatric condition, behavioral or neuropsychiatric problems are overrepresented in this group (6).

The pathophysiology behind constipation in children without neurogenic bowel dysfunction or anatomic malformations is unclear, but the condition is linked to behavioral issues and/or detrusor overactivity (7).

Several explanations have been given to the association of constipation and bladder-related problems such as enuresis and daytime incontinence:

1. The bladder and rectum have overlapping innervation and central nervous representation (8).
2. The direct anatomical relations in the pelvis ensure that a dilated rectum will distort the bladder, presumably causing detrusor overactivity (9).
3. Bladder and bowel problems are both linked to behavioral issues and neuropsychiatric conditions (6).

There is no simple, unequivocal test to diagnose constipation. Instead it is recommended that the anamnestic Rome III criteria be used, at least in the research setting (10). The so far best way to objectivize a suspicion of constipation is by ultrasonographic measurement of the horizontal rectal diameter behind the bladder (11–13). If this diameter is above 30 mm in a child who is not sensing any urge to defecate then constipation is probable (12).

The gold standard for the detection of detrusor overactivity is cystometry (14). However, this is an invasive examination that is not defensible in the evaluation of children with nocturnal enuresis, unless there are valid suspicions of neurogenic bladder (15). Instead, the clinician looks for indirect signs of detrusor overactivity in the case history and voiding chart data. Foremost of these signs are urgency symptoms, increased voiding frequency, and small voided volumes (16–18). If these are present in an enuretic child then it is assumed that detrusor overactivity is part of the picture. This may influence the choice of therapy.

Given that constipation is linked to detrusor overactivity, it may be assumed that constipated children would be more prone to urgency, increased voiding frequency, and/or low voided volumes. This should at least be the case if the anatomic mechanisms behind the bladder–bowel association mentioned above were correct. This has not been tested before. We chose to test this hypothesis in a group of children with enuresis, largely of the therapy-resistant kind. This group is suitable since among these children detrusor overactivity can safely be assumed to be common but not ubiquitous—i.e. many but not all of them can be assumed to have detrusor overactivity (4,5).

## Methods

This is a retrospective study on patients attending a tertiary care pediatric outpatient ward between 2004 and 2015. All patient files of children aged at least 6 years with enuresis and no daytime incontinence and no underlying neurologic or anatomic issues were reviewed. Patients who had, during standard evaluation, completed a voiding chart for at least 48 h, according to the specifications of the International Children’s Continence Society (14), and whose rectal diameter had been measured were included in the analysis.

During rectal diameter assessment the 6 MHz probe was placed approximately 2 cm above the symphysis at a 10–15-degree downward angle, as described in Joensson et al. (12). The cutoff between dilated and undilated rectum was set to 30 mm.

No antienuretic or laxative therapy was given during these examinations.

These examinations were performed during the baseline examinations of children recruited into several clinical studies, all of which were accepted by the Ethical Review Board of Uppsala University (2008/203, 2007/262, 2010/336). The research conformed to the provisions of the Declaration of Helsinki.

The central comparison in this study was the voiding frequencies of children with or without dilated rectum. The power calculations were made on the assumption that we wanted to have a 95% chance not to miss a true difference of one micturition/day. This yielded a minimum sample size of *≥*22 patients in both groups, i.e. with dilated and non-dilated rectum.

Comparisons between children with dilated and non-dilated rectum were made using the *t* test (normally distributed scale variables) or chi-square test (categorical data). The level of statistical significance was set at 95% (*p* < 0.05)

## Results

Altogether 74 children were eligible for inclusion. Their ages were 10.2 ± 2.8 years (range 6–15), and 12 of them were girls. Eight children, all of them boys, had a confirmed attention deficit hyperactivity disorder. All children had without success tried first-line antienuretic therapy, i.e. desmopressin and the enuresis alarm. Their anamnestic data, voiding chart data, and rectal diameters were obtained according to the methods described above (Table 1); 35 of the 74 children, i.e. 47.3%, were found to have a rectal diameter of 30 mm or more.

**Table 1.** Anamnestic data, voiding chart data, and rectal diameter.

Variable	*n*	Proportion, or mean ±1 SD
Urgency	69	54%
Previous daytime incontinence	74	28%
Constipated according to Rome III criteria	74	23%
Fecal incontinence	74	19%
Neuropsychiatric diagnosis	73	11%
Micturition frequency	72	6.1 ± 2.1
Average voided volumes, 1st morning void excluded	72	36 ± 15
Average voided volumes, 1st morning void included	72	39 ± 15
Maximum voided volumes, 1st morning void excluded	72	59 ± 23
Maximum voided volumes, 1st morning void included	72	70 ± 29
Nocturnal urine production during wet nights	71	102 ± 43
Enuresis volume	68	52 ± 29
Rectal diameter	74	33.2 ± 11.1 mm

All volume variables expressed as percentages of expected bladder capacity for age, according to the Koff–Hjälmås formula (19).

Children with and without rectal dilatation were compared (Table 2). All scale variables were found to be normally distributed. As can be seen, there were no statistically significant differences, although a trend towards slightly larger voided volumes among children with rectal dilatation could be noted. This trend was, however, not reflected by any reciprocal difference in micturition frequency.

**Table 2.** Anamnestic data.

Variable	Rectal diameter		*p* value
	>30 mm	≤30 mm	
Sex (girl/boy)	5.7/94.3	25.6/74.4	0.02
Urgency (y/n)	51.5/48.5	55.6/44.4	0.74
Previous daytime incontinence (y/n)	28.6/71.4	28.2/71.8	0.97
Rome III criteria positive (y/n)	28.6/71.4	17.9/82.1	0.28
Fecal incontinence (y/n)	25.7/74.3	12.8/87.2	0.16
Neuropsychiatric diagnosis (y/n)	17.6/82.4	7.7/92.3	0.20
Micturition frequency	6.1 ± 2.3	6.1 ± 1.9	0.97
Average voided volumes, 1st morning void excluded	37 ± 16	35 ± 14	0.54
Average voided volumes, 1st morning void included	41 ± 17	37 ± 13	0.24
Maximum voided volumes, 1st morning void excluded	63 ± 23	56 ± 23	0.26
Maximum voided volumes, 1st morning void included	77 ± 31.1	64 ± 25	0.06
Nocturnal urine production during wet nights	104 ± 39	100 ± 48	0.72
Enuresis volume	51 ± 31	53 ± 29	0.79

Comparison between children with (*n* = 35) and without (*n* = 39) increased rectal diameter.

Data presented are either proportions (percentages) or mean ±1 SD. All volume variables expressed as percentages of expected bladder capacity for age, according to the Koff–Hjälmås formula (19).

No anamnestic variable differed between the two groups, but it was found that boys were significantly more likely to have dilated rectum than girls (*p* = 0.02).

## Discussion

We tested whether rectal distension in enuretic children was reflected by anamnestic and voiding chart data and found this not to be the case. Thus, we found no indirect evidence that constipation was linked to detrusor overactivity in this group of children who are known to be prone to both conditions.

How can we explain this lack of differences? If constipation caused detrusor overactivity, then this should be reflected in voiding chart data. This leads to the tentative conclusion that in this patient group either detrusor overactivity causes the constipation or, more likely, they both share a common underlying mechanism. This common factor could probably best be explained by the partly shared neurology of the rectum and the lower urinary tract (8).

On the other hand, rectal distension may also cause detrusor inhibition (20), and constipation is known to be linked to voiding dysfunction—i.e. sphincter contraction during voiding—as well (7). Furthermore, behavioral issues may be relevant in this context. It is known that neuropsychiatric conditions are overrepresented among children with bladder and/or bowel conditions such as enuresis, daytime incontinence, voiding postponement, and fecal incontinence (6). Our data are too limited regarding behavioral or psychiatric aspects for any conclusions to be drawn but do point in the same direction. It may thus be speculated that a subgroup of enuretic children with behavioral issues habitually postpone both defecation and micturition, resulting in constipation without increased voiding frequency or small voided volumes.

The picture is probably mixed and complex, with anatomic, neurologic, and behavioral factors influencing the voiding habits in conflicting directions.

There are several drawbacks and uncertainties with this study, the most important one being the retrospective nature of the investigation. It should thus be regarded as hypothesis-generating rather than hypothesis-proving. We do, however, believe that the population studied was large enough: if there are any clinically relevant true differences in micturition habits between enuretic children with and without rectal dilatation we would have found them.

Another problem is that we, in common with almost everyone studying enuresis, do not know which of the children actually had detrusor overactivity, since we—for ethical reasons—did not perform any invasive urodynamic investigations. In fact, it may be questioned how adequate voiding charts are in the detection of detrusor overactivity. The evidence for the link between detrusor overactivity, increased voiding frequency, and small voided volumes is quite weak and mostly based on studies on adults (16,17). Maybe the voiding chart reflects behavior more than bladder function. Furthermore, even the link between urgency and detrusor overactivity is not absolute (17,21), especially not in children, who may find it difficult to detect or describe this symptom.

An alternative way to infer whether an enuretic child has underlying detrusor overactivity is if anticholinergic medication—a recognized second-line antienuretic therapy—is successful. However, since the study was retrospective and the choice of treatment for the individual patient was not based on a standardized protocol, we decided not to pursue this line of investigation further.

One comparison that did show a statistically significant difference was not included in our hypothesis but still deserves mention: the one between sexes. A lower proportion of the girls had an increased rectal diameter in comparison with the boys. One may speculate whether this difference, if confirmed by prospective studies, means that urodynamic mechanisms (as opposed to polyuria) are more important in enuresis pathogenesis in boys. It should be remembered that we do not know why enuresis is more common in boys in the first place.

In conclusion, in this preliminary pilot study we found no clear differences in voiding chart data or anamnestic signs of detrusor overactivity between enuretic children with and without rectal dilatation. We plan to address this issue in an upcoming, prospective study looking at enuretic as well as non-enuretic children, taking behavioral variables into account.
